# m6A Methylation Regulator RBM15-Mediated Upregulation of ITGBL1 mRNA Stability Aggravates Colon Adenocarcinoma Progression by Remodeling the Tumor Microenvironment

**DOI:** 10.5152/tjg.2025.24068

**Published:** 2025-01-13

**Authors:** Jie Zhu, Dengliang Liu, Yingying Zou

**Affiliations:** Department of Gastrointestinal and Thoracic Surgery, Jiulongpo People’s Hospital, Chongqing, China

**Keywords:** Integrin β-like 1 protein, RNA-binding motif protein-15, colon adenocarcinoma, macrophage, CD8+ T cells

## Abstract

**Background/Aims:**

Colon adenocarcinoma (COAD) is a prevalent malignant tumor of the digestive system. Previous research has indicated that RNA N6-methyladenosine (m6A) methyltransferase RNA-binding motif protein-15 (RBM15) is involved in various cancers. We aimed to investigate the function of RBM15 in COAD progression and its underlying molecular mechanism.

**Materials and Methods:**

TIMER and UALCAN databases were applied to analyze the relationship between COAD and Integrin β-like 1 protein (ITGBL1) or RBM15. RT-qPCR and Western blot were used to analyze ITGBL1, M2-type macrophage markers, EMT-related markers, and RBM15 expression. CCK-8, colony formation, and transwell experiments detected cell viability, proliferation, migration, and invasion. The effect of ITGBL1 on COAD tumor growth was examined using a xenograft tumor model. The effects of COAD cells on macrophage polarization and the proliferation and apoptosis of CD8^+^ T cells were analyzed using flow cytometry analysis. Relationships between RBM15 and ITGBL1 were validated using MeRIP and dual-luciferase reporter assay.

**Results:**

ITGBL1 and RBM15 contents were elevated in COAD. ITGBL1 knockdown could hinder COAD cell proliferation, migration, invasion, M2-type macrophage polarization, and lymphocyte immunity. Meanwhile, the lack of RBM15 dampened tumor growth *in vivo. *Mechanistically, RBM15 could increase ITGBL1 expression by m6A methylation.

**Conclusion:**

RBM15 could promote COAD progression by regulating ITGBL1 mRNA stability, providing a promising biomarker and a potential target for COAD.

**Main Points**

- ITGBL1 content was enhanced in colon adenocarcinoma.
- ITGBL1 knockdown could repress colon adenocarcinoma cell growth and immune response.
- RBM15 sustained ITGBL1 expression.

## Introduction

As a prevalent gastrointestinal malignancy of epithelial origin in the colon, colon adenocarcinoma (COAD) has been recognized as a major cause of cancer-related death globally.^1^ However, the understanding of mechanisms and therapeutic options is still limited. Significant advances in conventional treatments, including screening, early diagnosis, surgical resection, chemotherapy, adjuvant therapy, and targeted drugs, have recently achieved remarkable clinical gains.^2-5^ Still, most sufferers with advanced or metastatic COAD often have a poor prognosis.^6^ Therefore, the exploration of effective intervention measures is crucial. This project aimed to conduct insightful research into the mechanisms involved in COAD and find effective therapeutic strategies, which are of great significance in alleviating the condition of COAD patients and improving their survival rate and quality of life.

Nowadays, cancer immunotherapy has received increasing attention for treating recurrent or metastatic cancer.^7^ A recent study has suggested that integrin β-like 1 protein (ITGBL1, also known as TIED) was aberrantly upregulated in tumors from patients resistant to anti-PD1 based on RNAseq analysis.^8^ Furthermore, ITGBL1 is a secreted protein that allows melanoma cells to escape immune surveillance by repressing natural killer (NK) cell cytotoxicity.^9^ Dysregulated ITGBL1 could boost tumor cell invasion and metastasis via different pathways.^10,11^ During colon cancer research, the high expression of ITGBL1 promotes tumor cell proliferation and migration.^12,13^ Besides, earlier literature suggested that ITGBL1 expression data was continuously increasing in colorectal cancer (CRC) from normal to distant organ metastasis.^14^metastasis. The above data implied that ITGBL1 could partake in the formation of the tumor microenvironment (TME), but its mechanism in CRC is still blurry.

N6-methyladenosine (m6A) is a common internal mRNA modification that is a key regulator of gene expression.^15^ In mammalian cells, m6A modification has reversible and dynamic properties, whose balance is orchestrated by three different protein complex types, containing methyltransferases (“writers”), demethylation (“erasers”), and m6A binding proteins (“readers”).^16^ As an important methyltransferase, RNA-binding motif protein-15 (RBM15) has been identified to be significantly upregulated in COAD patients.^17^ Beyond that, RBM15 could facilitate CRC cell growth and metastasis by regulating MyD88 stability.^18^ Moreover, RBM15-mediated m6A modification of KLF1 mRNA increased KLF1 stability and boosted CRC cell proliferation and migration.^19^ Besides, RBM15 expression was positively related to immune infiltrating cells in various tumors.^20,21^ Herein, bioinformatics analysis revealed the binding between RBM15 and ITGBL1 in COAD cells. Hence, we aimed to illuminate whether RBM15 could control COAD progression by regulating ITGBL1 mRNA stability.

## Materials and Methods

### Clinical Samples and Cell Culture

Forty-five COAD tissues and 45 paracancerous tissues from the same patients were collected from Jiulongpo People’s Hospital hospital. Inclusion criteria were as follows: (1) patients older than 18 years; (2) patients with pathologically confirmed COAD; (3) patients who had not received any related treatment before surgery; and (4) patients with registration data included in our hospital. The exclusion criteria were as follows: (1) patients without complete clinical treatment history; (2) patients who had received radiotherapy or chemotherapy; (3) patients who had a history of another malignant tumor other than COAD; and (4) patients who suffered from congenital or acquired immunodeficiency diseases. Immediately after the operation, these tissues were stored at −80°C until use. All participants gave written informed consent. The detailed clinical characteristics of patients are described in Table 1. The experiment sample collection was authorized by the Ethics Committee of Jiulongpo People’s Hospital (approval number: 2024022, date: November 13, 2023).

Colon adenocarcinoma cell lines (DLD1, CL-0074; HCT15, CL-0097; Procell, Wuhan, China) were cultivated in corresponding media (CM-0074, CM-0097, Procell) at 37°C with 5% CO_2_. Meanwhile, the other 2 COAD cell lines (Lovo, CC-Y1319; SW620, CC-Y1502, Elisakits, Shanghai, China) were incubated in corresponding media (CC-Y1319M, CC-Y1502M, Elisakits). In addition, a normal human colon mucosal epithelial cell line (NCM460, INCELL Corporation, San Antonio, TX, USA) was routinely maintained in M3 TM Base medium (M310A; INCELL Corporation).

### Real-Time Quantitative Polymerase Chain Reaction

Based on the Trizol reagent (Invitrogen, Paisley, Scotland, UK), total RNA from clinical samples and cell lines was prepared. After reversing transcribing into cDNA first strands with the Maxima H Minus cDNA Synthesis Master Mix kit (Invitrogen) using 1 μg RNA, amplification reaction was performed with SYBR Green SuperMix (Roche, Basel, Switzerland). β-actin was employed as an internal control. RNA expression fold changes were determined with the 2^–ΔΔCt^ method. The primers used are shown in Table 2.

### Western Blot Assay

To collect protein samples from tissues and cells, RIPA lysis buffer (CST, Danvers, MA, USA) and phosphatase inhibitors were utilized. After being separated by SDS-PAGE, samples were transferred onto PVDF membranes. Then, membranes were labeled with the following primary antibodies: ITGBL1 (CST, #34971, 1:1000), E-cadherin (ab40772, Abcam, Cambridge, MA, USA, 1:1000), N-cadherin (ab76011, Abcam, 1:5000), Vimentin (ab92547, Abcam, 1:1000), CD163 (CST, #93498, 1:1000), CD206 (CST, #24595, 1:1000), Interleukin-10 (IL-10; CST, #12163, 1:1000), RBM15 (CST, #60386, 1:1000), and β-actin (ab8227, Abcam, 1:1000) overnight at 4°C, followed by hybridization with a secondary antibody for 2 hours. Finally, the immunoreactive signals were visualized using ECL reagent (Solarbio, Beijing, China) and analyzed using Image J software.

### Cell Transfection

Briefly, HEK293T cells (CL-0005, Procell) at about 80% confluency were transfected with lentivirus packaging plasmids and GV248-shRNAs human ITGBL1 (sh-ITGBL1#1/#2) or RBM15 (sh-RBM15#1/#2)/GV248-target genes human ITGBL1 cDNA (NM_001271756.2) or RBM15 cDNA (NM_022768.5) constructs. In parallel, the GV248 lentiviral empty vector was applied as negative controls (sh-NC or vector) and transfected into HEK293T cells based on the above method. Forty-eight hours post transfection, secreted virus particles in the medium were harvested and filtered, followed by infecting tumor cells in the presence of polybrene. Finally, stable cell lines were selected using puromycin.

### CCK-8

In this experiment, the assessment of COAD cell viability was performed. Briefly, 2 × 10^4^ cells were mixed with 10 μL CCK-8 (Dojindo, Kumamoto, Japan) at the designated culturing period. Four hours later, the absorbance was recorded with a microplate reader at 450 nm.

### Colony Formation Assay

Five hundred COAD cells were cultured for 14 days at 37°C. Then, visible colonies were fixed and stained. Finally, cell colonies with >50 cells were counted.

### Transwell Assay

For migration experiments, 5 × 10^4^ cells were planted in 200 μL medium into transwell chambers (Chemicon, Temecula, CA, USA) in the insert of a 24-well plate. Meanwhile, the lower compartments were filled with 600 μL of cell culture medium with 10% FBS. After 24 hours, the migrated cells in lower filters were fixed, stained, and counted using a microscope (Leica, Wetzlar, Germany). For the invasion assay, 1 × 10^5^ cells were introduced into the upper counterpart with Matrigel (BD Biosciences, Heidelberg, Germany).

### Tumor Xenograft Assay

In general, BALB/C male nude mice were provided by Slaike Jingda Laboratory (Hunan, China), followed by random splitting into 2 groups (n = 5 for each group). Subsequently, 5 × 10^6^ SW620 cells transfected with sh-NC or sh-ITGBL1 were subcutaneously injected into 5- to 6-week-old mice. During this period, tumor size was examined weekly. After five injections, all mice were euthanized for tissue imaging, weight, and obtainment. Then, immunohistochemical (IHC) staining was conducted to detect target gene-positive expression in dissected tissues. Mouse xenograft procedures were authorized based on the Animal Ethics Committee of Jiulongpo People’s Hospital (approval number: 2024023, date: November 13, 2023).

### Flow Cytometry

Firstly, 5 × 10^5^ THP1 cells were differentiated into macrophage-like cells (M0 macrophages) with 100 ng/mL PMA for 48 hours. For conditioned media (CM) preparation, transfected COAD cells that reached 80% confluency were washed and changed to a medium without fetal bovine serum (FBS). Then, CM was collected and filtered at 0.2 μm after 48 hours of incubation. For COAD/THP1-M0 co-culture, THP1-M0 cells were treated with the corresponding 50% (v/v) CM of COAD cells for 48 hours, followed by PBS washing and fresh medium incubation for 24 hours.

To check the macrophage polarization, co-cultured cells were harvested and incubated with FITC (Beyotime, Shanghai, China)-conjugated anti-human CD206^+^ (Proteintech, Rosemount, MN, USA) in a staining buffer before analysis using flow cytometry.

### T-Cell Proliferation and Apoptosis

CD8^+^ T cells from peripheral blood mononuclear cells (PBMCs) were purified by EasySep™ Direct Human CD8^+^ T cell Isolation Kit (STEMCELL, Vancouver, Canada). Then, 1 × 10^6^ CD8^+^ T cells were resuspended in culture medium and added to pre-adherent COAD cells at an effector-to-target cell ratio of 3 : 1. After being collected, CD8^+^ T-cell proliferation or apoptosis were incubated with CFSE (Invitrogen) and Annexin V-FITC and PI (Beyotime), respectively. At last, results were assessed using a flow cytometer.

### Methylated RNA Immunoprecipitation (MeRIP)

To examine m6A modification of ITGBL1 mRNA, total RNAs were purified using TRIzol and then fragmented to about 300 bp using an ultrasonic cell disruptor. Subsequently, one-ninth of the mRNA was kept as an “Input,” whereas the rest was immunoprecipitated with IgG or m6A antibody (MABE1006, Millipore). After being washed and eluted, these immunoprecipitated RNAs or input RNAs from each sample were determined with real-time quantitative polymerase chain reaction (RT-qPCR).

### Dual-Luciferase Reporter Assay

To further analyze the relationship between RBM15 and ITGBL1, this experiment was performed in Lovo and SW620 cells. In short, the ITGBL1 sequence containing the potential m6A site was cloned downstream of the pGL3 luciferase reporter plasmid (Promega, Madison, WI, USA), generating ITGBL1^WT^. Meanwhile, mutations (ITGBL1^MUT^) were performed in the binding sites. Subsequently, 3 × 10^4^ COAD cells were co-transfected with a mixture of 800 ng construct and 20 pmol shRNA. After 48 hours, luciferase activities were detected with a dual-luciferase reporter assay system (Promega).

### Statistical Analysis

Statistical analyses were performed using GraphPad Prism7 software (GraphPad, San Diego, CA, USA). Data were shown as mean ± standard deviation (SD). Student’s *t*-test or one-way ANOVA with Tukey’s test was carried out for comparisons. Statistical significance was set at *P* < .05.

## Results

### ITGBL1 is Overexpressed in COAD

**Figure 1.**
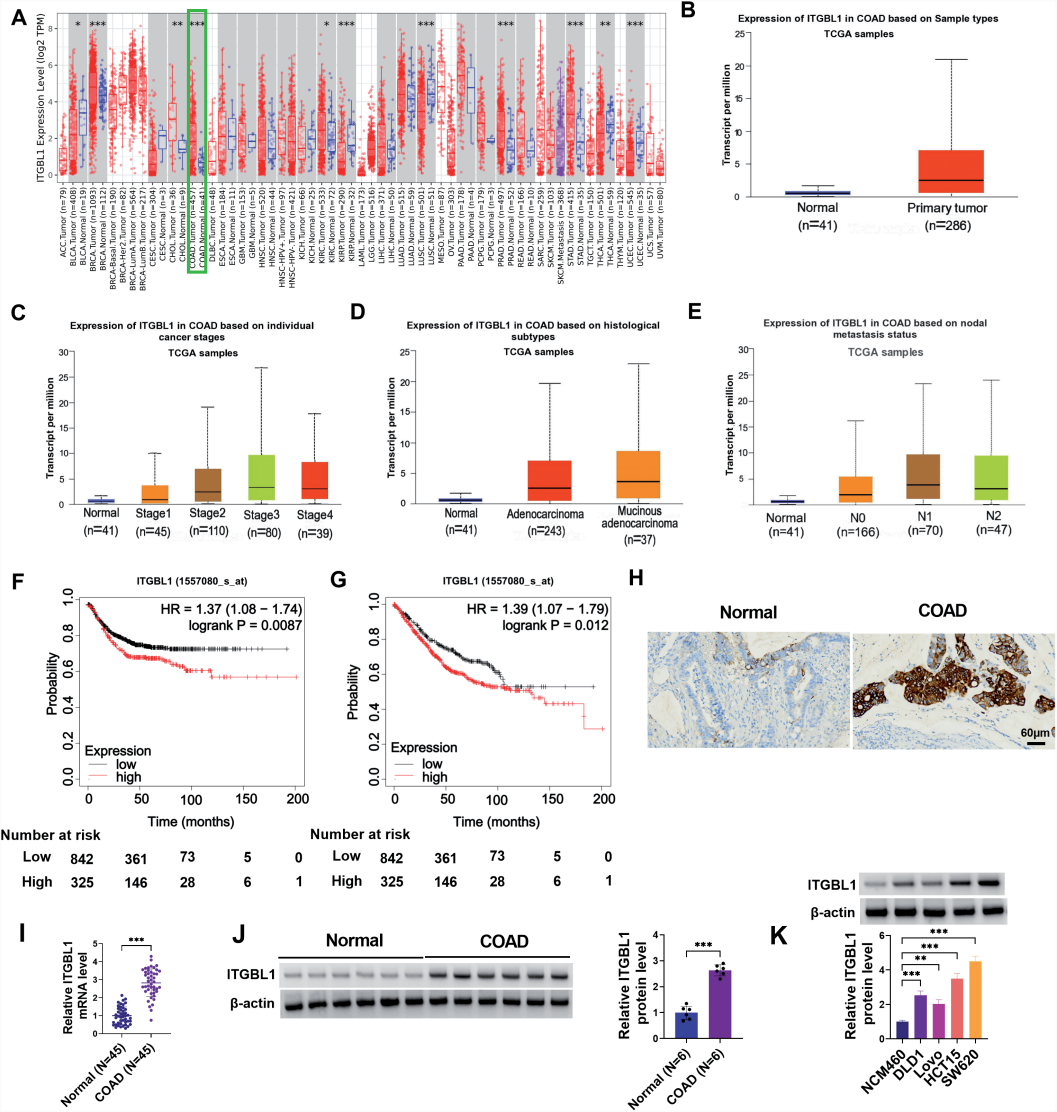
Expression pattern of ITGBL1 in COAD. (A) The TIMER database was used to display the differential expression of ITGBL1 in the Cancer Genome Atlas (TCGA) pan-cancer dataset. (B) UALCAN database showed ITGBL1 expression in COAD based on sample types TCGA samples. (C and E) The UALCAN database shows the association between ITGBL1 expression and COAD individual cancer stage, nodal metastatic status, and histologic subtype. (F and G) KM plotter database was used to analyze the relationship between ITGBL1 expression and RFS and OS of COAD. (H) Immunohistochemical observation of ITGBL1 expression in normal and COAD tissues. (I) RT-qPCR assay was used to detect the expression level of ITGBL1 in 45 normal tissues and 46 COAD tumor tissues. (J and K) Western blot analysis of ITGBL1 protein level in normal tissues, COAD tumor tissues, NCM460 cell line, and COAD cell lines (DLD1, Lovo, HCT15, and SW620). **P < .01, ***P < .001.

To assess the effects of ITGBL1 on the genesis of human tumors, its mRNA level was analyzed in 66 types of cancer based on the TCGA database. The TIMER database showed that ITGBL1 was highly expressed in many cancers, including COAD (Figure 1A). This indicated that it may act as a key oncogene in 11 cancers. Furthermore, UALCAN databases exhibited a significant upregulation of ITGBL1 in COAD samples (Figure 1B). Then, the relationship between ITGBL1 content and different clinical parameters was further evaluated. Data showed that ITGBL1 was clearly increased in individual cancer stages, histologic subtypes, and nodal metastasis status of COAD (Figure 1C-E). Based on ITGBL1 expression level, patients were classified into high-ITGBL1 or low-ITGBL1 subgroups. Subsequently, we found that patients with higher ITGBL1 expression had shorter overall survival (OS) rates and relapse-free survival (RFS) rates in the TCGA cohort (Figure 1F and G), implying that ITGBL1 was a promising candidate biomarker for predicting the relapse of COAD sufferers. Beyond that, the ITGBL1 positive expression rate was upregulated in COAD tissues (Figure 1H). Consistent with the above results, a dramatic increase in ITGBL1 was observed in COAD samples (Figure 1I and J). To probe the association of ITGBL1 expression with clinicopathologic features, the 45 patients with COAD were then classified in Table 1. Results showed that ITGBL1 expression was associated with distant metastasis and clinical stage (*P *< .05). Moreover, we further verified that ITGBL1 was highly expressed in COAD cell lines (DLD1, Lovo, HCT15, and SW620) compared with the NCM460 cell line (Figure 1K). Given that ITGBL1 displayed the highest fold change in Lovo and SW620 cells, these 2 cell lines were selected for the following study. Collectively, the elevation of ITGBL1 expression indicated poor clinical outcomes in patients with COAD.

### Knockdown of ITGBL1 Could Block COAD Cell Malignant BehaviorsIn Vitro

**Figure 2.**
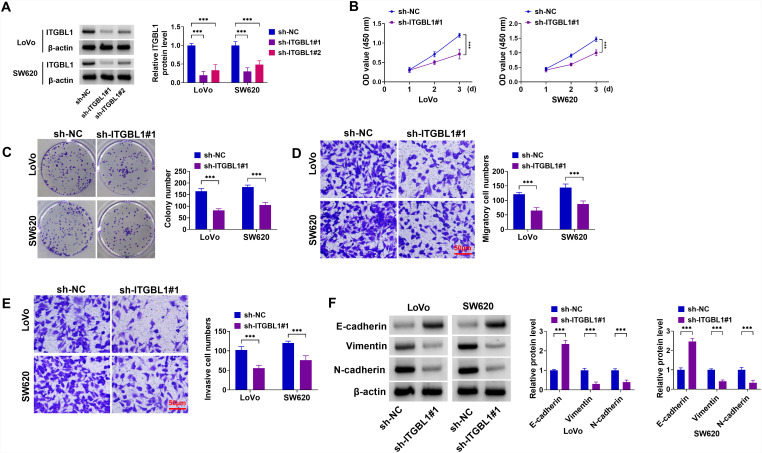
Effects of ITGBL1 downregulation in COAD cell proliferation, migration, invasion, and EMT. Lovo and SW620 cells were transfected with sh-NC, sh-ITGBL1#1, or sh-ITGBL1#2. (A) ITGBL1 protein level was determined using western blot in transfected Lovo and SW620 cells. (B and C) Cell viability and proliferation were measured in transfected Lovo and SW620 cells using CCK-8 and colony formation assays. (D and E) Cell migration and invasion were assessed using Transwell assays in transfected Lovo and SW620 cells. (F) Western blot analysis of E-cadherin, Vimentin, and N-cadherin protein levels in transfected Lovo and SW620 cells. ***P < .01.

Subsequently, the role of ITGBL1 knockdown in Lovo and SW620 cells was analyzed. First of all, the ITGBL1 protein level was significantly decreased in sh-ITGBL1#1 or sh-ITGBL1#2-transfected COAD cells compared with cells with sh-NC (Figure 2A). Specifically, sh-ITGBL1#1 (sh-ITGBL1), hence it was selected for subsequent experiments. Then, cell viability and proliferation were markedly repressed by ITGBL1 downregulation in Lovo and SW620 cells (Figure 2B and C). In parallel, Transwell assays displayed that the deficiency of ITGBL1 could remarkably constrain cell migration and invasion in Lovo and SW620 cells (Figure 2D and E). In terms of EMT, western blot assay presented that ITGBL1 silencing improved E-cadherin and declined N-cadherin and Vimentin levels in Lovo and SW620 cells (Figure 2F), indicating the inhibitory role of ITGBL1 knockdown on EMT. Together, these data findings implied that ITGBL1 absence could diminish COAD cell progression.

### ITGBL1 Inhibition Could Impair COAD Cell Growth* In Vivo*

**Figure 3.**
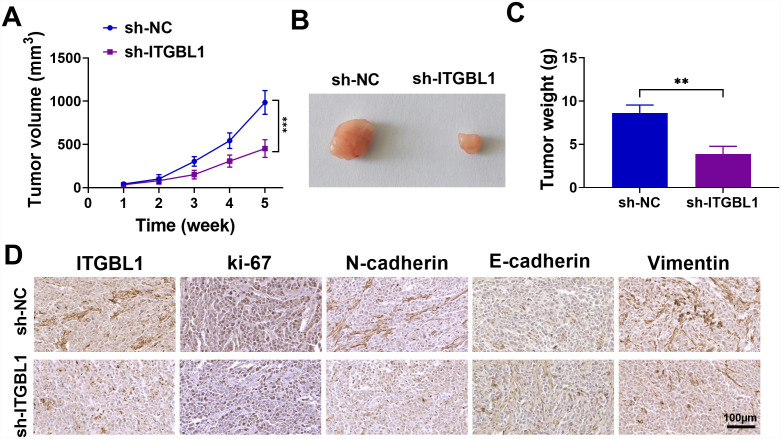
Inhibiting ITGBL1 impaired COAD cell growthin vivo. SW620 cells stably infected with sh-NC or sh-ITGBL1 were respectively subcutaneously inoculated into mice. (A and B) Tumor growth curve of xenografts and representative images of the tumors at the end of the experiment were presented. (C) Tumor weight was measured. (D) IHC staining was performed to measure the positive expression of ITGBL1, Ki-67, N-cadherin, E-cadherin, and Vimentin in xenografts. **P < .01, ***P < .001.

To further validate the in vivo effect of ITGBL1 on COAD cell malignancy*, *we used a COAD xenograft mouse model. Unsurprisingly, tumors in the sh-ITGBL1 group were smaller in size and lighter in weight compared to the sh-NC group (Figure 3A-C), suggesting ITGBL1 absence repressed tumor growth in vivo. Meanwhile, IHC staining in tissue samples found that ITGBL1, Ki-67, N-cadherin, and vimentin positive expression rates were suppressed in the sh-ITGBL1 group, whereas E-cadherin was elevated (Figure 3D). Overall, ITGBL1 knockdown could effectively repress COAD cell growth in vivo.

### ITGBL1 Knockdown Relieved Immune Escape

**Figure 4.**
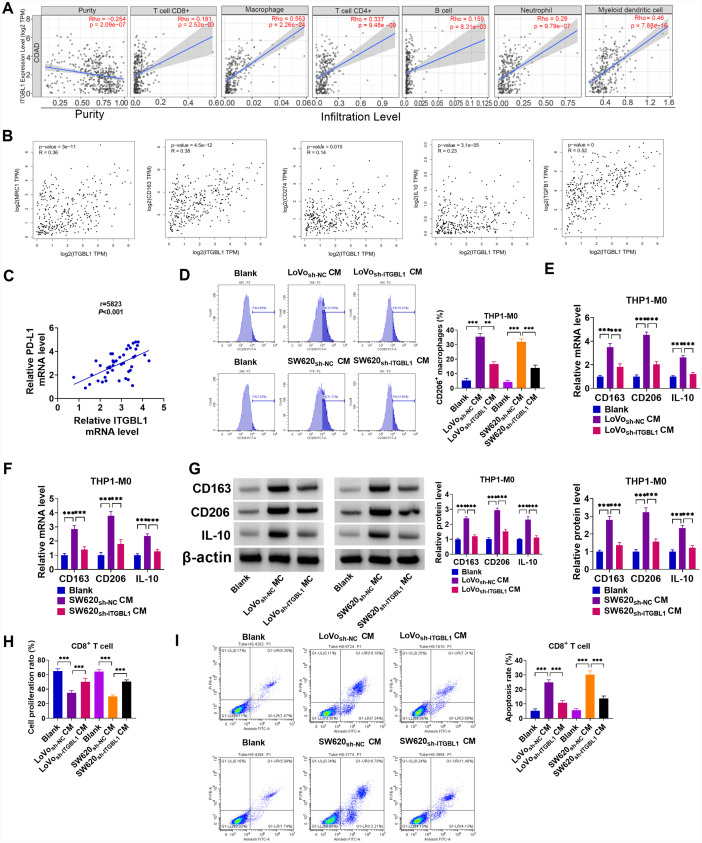
ITGBL1 regulates the immune response to cancer. (A) TIMER database displayed the correlation of ITGBL1 with tumor-infiltrating immune cells (CD8^+^ T cells, macrophages, CD4^+^ T cells, B cells, neutrophils, and myeloid dendritic cells) in COAD. (B) GEPIA database exhibited correlation of ITGBL1 with tumor-associated M2-type macrophage markers (MRC1, CD163, IL-10, and TGFB1) and PD-L1 (CD274) expression in COAD. (C) Expression association between ITGBL1 and PD-L1 in COAD tissues was evaluated using Pearson correlation analysis. (D-G) Lovo and SW620 cells were transfected with sh-NC or sh-ITGBL1. Then, CM of transfected Lovo and SW620 cells were co-culture with isolated macrophages (THP1-M0). (D) The proportion of CD206^+^ positive cells was measured using flow cytometry. (E and F) CD163, CD206, and IL-10 mRNA levels were detected using RT-qPCR. (G) CD163, CD206, and IL-10 protein levels were assessed using a western blot assay. (H and I) Effector CD8^+^ T cells were co-cultured with indicated target Lovo and SW620 cells. (H) CD8^+^ T cell proliferation was examined using CFSE staining. (I) CD8^+^ T cell apoptosis was monitored by flow cytometry assay. **P < .01, ***P < .001.

A previous study has suggested that ITGBL1 is a novel immunomodulator that promotes tumor development by inhibiting the cytotoxicity of NK cells.^9^ Subsequently, we further analyzed the correlation between ITGBL1 and different tumor-infiltrating immune cells according to the TIMER database. Data showed that ITGBL1 expression in COAD was positively associated with CD8^+^ T cells, macrophages, CD4^+^ T cells, B cells, neutrophils, and myeloid dendritic cells (Figure 4A). Meanwhile, GEPIA database analysis displayed that ITGBL1 expression was positively related to M2-type macrophage markers (MRC1, CD163, IL-10, and TGFB1) and programmed death ligand 1 (PD-L1; also known as CD274) in COAD (Figure 4B). These data implied that high ITGBL1 expression in M2 macrophage-rich TME could restrict anti-tumor immunity. As expected, ITGBL1 content was positively associated with PD-L1 expression in COAD clinical samples (Figure 4C). Subsequently, to further identify whether COAD cell-triggered polarization macrophages might be reprogrammed by ITGBL1, isolated THP1-M0 were co-cultured with the CM of sh-NC or sh-ITGBL1-transfected Lovo and SW620 cells, respectively. Moreover, the proportion of M2 marker CD206^+^ positive cells was remarkably decreased through ITGBL1 lack (Figure 4D). Analogously, RT-qPCR and western blot results exhibited that the downregulation of ITGBL1 could significantly reduce the mRNA levels and protein levels of CD163, CD206, and IL-10 (Figure 4E-G). Then, we further investigated the impact of ITGBL1 deficiency on the immune response of COAD cells to T cells. When sh-NC or sh-ITGBL1-transfected Lovo and SW620 cells were co-cultured with CD8^+^ T cells, CFSE staining confirmed that CD8^+^ T cell proliferation rate in Lovo_ sh-_ _ITGBL1_ CM and SW620_ sh-ITGBL1_ CM groups was clearly enhanced compared with the corresponding control groups (Figure 4H). In addition, flow cytometry results presented that the lack of ITGBL1 could effectively relieve the proportion of CD8^+^ T cell apoptosis (Figure 4I). Overall, these experiments suggested that targeting ITGBL1 inhibition could block M2-type macrophage polarization and improve lymphocytic immunity, resulting in anti-tumor properties in vitro.

### RBM15 Could Improve ITGBL1 Expression Through Methylation Modification

**Figure 5.**
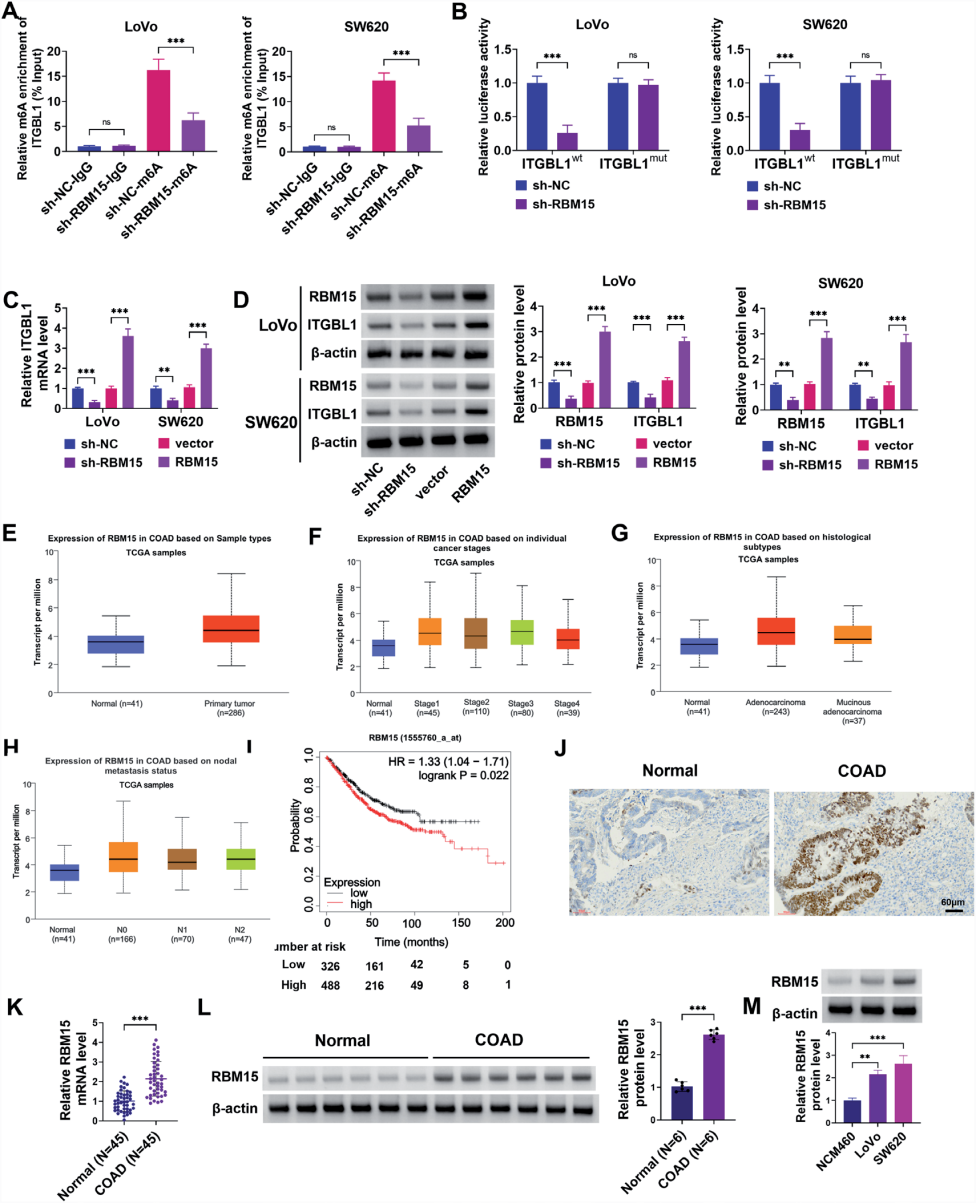
RBM15 stabilizes ITGBL1 expression through methylation modification. (A) Changes in the m6A methylation level of ITGBL1 after inhibition of RBM15 were analyzed by MeRIP-qPCR assay. (B) Their interaction was verified using a dual-luciferase reporter assay in Lovo and SW620 cells. (C) ITGBL1 mRNA level was measured in Lovo and SW620 cells transfected with sh-NC, sh-RBM15, vector, or RBM15 using RT-qPCR. (D) RBM15 and ITGBL1 protein levels were assessed in Lovo and SW620 cells transfected with sh-NC, sh-RBM15, vector, or RBM15 using western blot. (E-H) The UALCAN database exhibited the association between RBM15 expression and COAD sample types, individual cancer stages, nodal metastatic status, and histologic subtypes. (I) The KM plotter database was applied to analyze the relationship between RBM15 expression and the OS of COAD. (J) IHC staining was used to detect the positive expression of RBM15 in normal and COAD tissues. (K) RBM15 mRNA level was determined in 45 normal tissues and 46 COAD tumor tissues using RT-qPCR. (L and M) Western blot analysis of RBM15 protein levels in normal tissues, COAD tumor tissues, NCM460 cell line, and COAD cell lines (Lovo and SW620). **P < .01, ***P < .001.

Based on the SRAMP website, there are m6A modification sites on the mRNA of ITGBL1. Subsequently, ITGBL1 was found to possess binding sites with RBM15 according to the RBPsuite website. Hence, we inferred that RBM15 could affect ITGBL1 m6A methylation. Then, the lack of RBM15 could clearly decrease ITGBL1 m6A modification levels in Lovo and SW620 cells (Figure 5A). Consistently, the luciferase activity of ITGBL1 was also significantly reduced by the knockdown of RBM15 in Lovo and SW620 cells (Figure 5B). ITGBL1 mRNA levels were hindered through RBM15 silencing and remarkably increased through RBM15 overexpression (Figure 5C). Meanwhile, western blot analysis found that RBM15 and ITGBL1 protein levels were strikingly inhibited when RBM15 was depleted, whereas their expressions were enhanced after RBM15 introduction in Lovo and SW620 cells (Figure 5D). In addition, the UALCAN database exhibited that RBM15 was elevated in the sample types, individual cancer stages, nodal metastatic status, and histologic subtypes of COAD (Figure 5E-H). Simultaneously, in the TCGA Cohort, the OS rate was shorter in patients with higher RBM15 expression in COAD (Figure 5I). As expected, IHC staining observed a significantly higher rate of positive RBM15 expression in COAD tissues than in normal tissues (Figure 5J). Similarly, RBM15 mRNA and protein levels were augmented in COAD samples compared with normal samples (Figure 5L and K). Apart from that, RBM15 was highly expressed in Lovo and SW620 cells (Figure 5M). Collectively, the above data illuminated that RBM15 could promote ITGBL1 expression by regulating RNA m6A modification in COAD cells.

### Downregulation of RBM15 Repressed COAD Growth and Metastasis by Regulating ITGBL1

**Figure 6.**
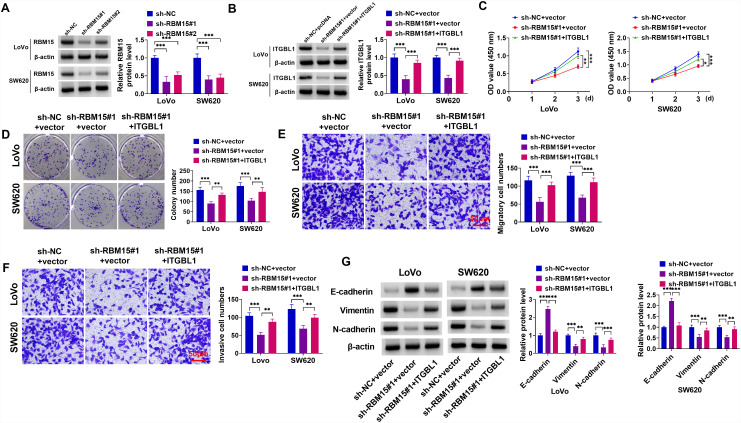
RBM15/ITGBL1 regulated COAD cell proliferation, migration, invasion, and EMT. (A) The transfection efficiency of sh-RBM15#1 or sh-RBM15#2 in Lovo and SW620 cells was measured using western blot. (B-G) Lovo and SW620 cells were transfected with sh-NC, sh-RBM15#1, sh-RBM15#1+ vector, or sh-RBM15#1+ITGBL1. (B) Western blot analysis of RBM15 protein levels in transfected Lovo and SW620 cells. (C and D) CCK-8 and colony formation assays were performed to assess cell viability and proliferation. (E and F) Transwell assays were conducted to measure cell migration and invasion. (G) E-cadherin, Vimentin, and N-cadherin protein levels were determined using western blot. **P < .01, ***P < .001.

Next, to further explore whether ITGBL1 could mediate the function of RBM15 in COAD, we performed a functional rescue experiment *in vitro*. At first, the knockdown efficiency of sh-RBM15#1 or sh-RBM15#2 in Lovo and SW620 cells was detected and exhibited in Figure 6A. Among them, the transfection efficiency of sh-RBM15#1 (sh-RBM15) was significant, so it was selected for the following experiments. Co-transfection of ITGBL1 could evidently counteract the repression of RBM15 knockdown on ITGBL1 protein levels in Lovo and SW620 cells (Figure 6B). After that, RBM15 silencing could attenuate Lovo and SW620 cell viability and proliferation, which was partly reversed by ITGBL1 upregulation (Figure 6C and D). Beyond that, RBM15 silencing-mediated migration and invasion suppression was significantly relieved by ITGBL1 overexpression (Figure 6E and F). Besides, RBM15 deficiency-mediated EMT inhibition was significantly ameliorated by ITGBL1 overexpression in COAD cells, as depicted by lower E-cadherin, and higher Vimentin and N-cadherin (Figure 6G). Overall, the lack of RBM15 could block COAD cell growth by modulating ITGBL1.

### RBM15/ITGBL1 Regulated Tumor Immune Response

**Figure 7.**
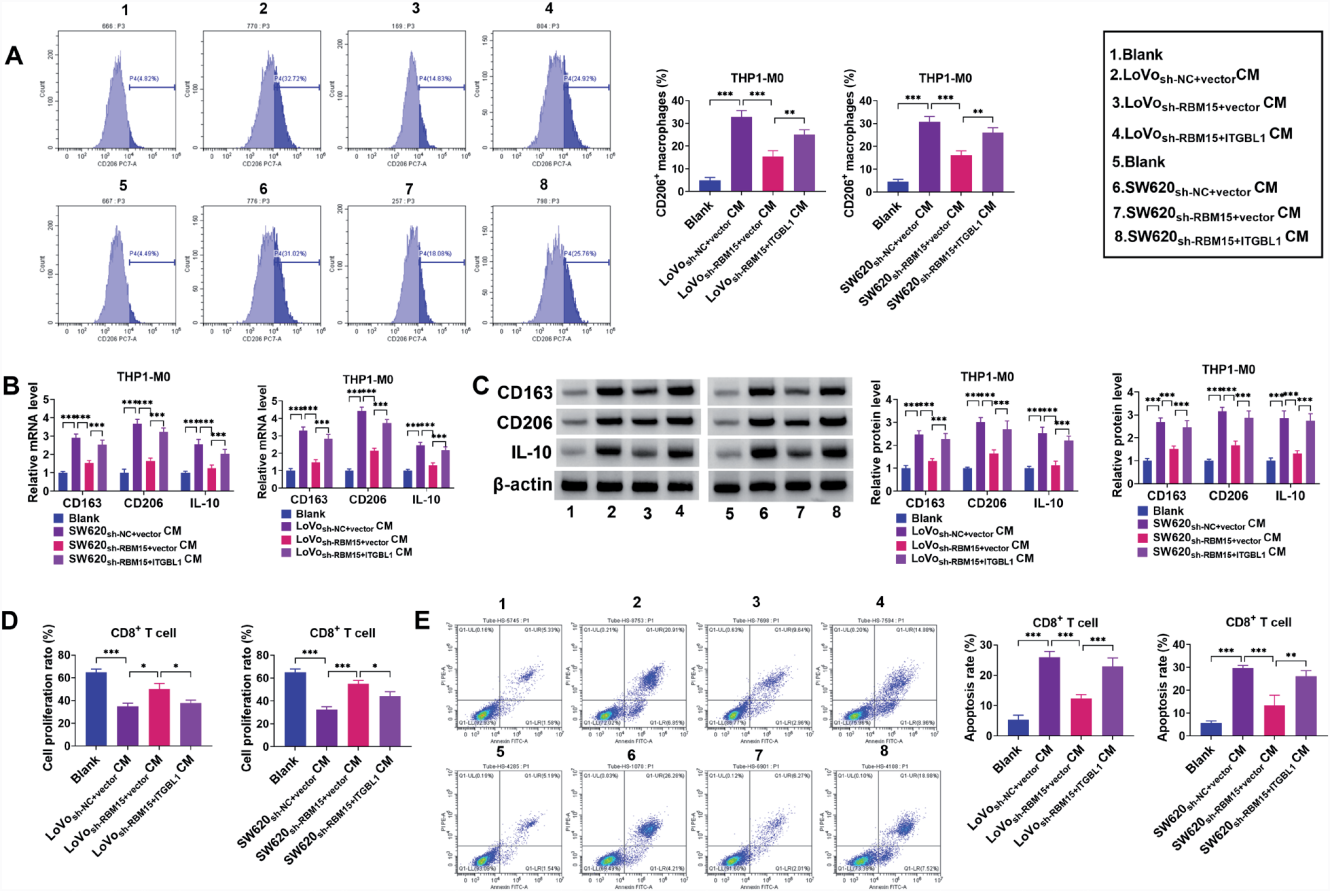
Overexpression of ITGBL1 reversed the regulatory effect of RBM15 knockdown on cancer immune response. (A-C) Lovo and SW620 cells were treated with Blank, sh-NC+ vector, sh-RBM15+ vector, or sh-RBM15+ ITGBL1. Then, CM of treated Lovo and SW620 cells were co-culture with isolated THP1-M0. (A) Flow cytometry analysis of the proportion of CD206^+^ positive cells. (B and C) The mRNA levels and protein levels of CD163, CD206, and IL-10 were detected using RT-qPCR and western blot. (D and E) Effector CD8^+^ T cells were co-cultured with indicated target Lovo and SW620 cells. (D) CFSE staining analysis of CD8^+^ T cell proliferation. (E) Flow cytometry analysis of CD8^+^ T cell apoptosis. *P < .05, **P < .01, ***P < .001.

Furthermore, the influences of RBM15 and ITGBL1 on COAD cell immune response were further investigated. As shown in Figure 7A, the proportion of M2 marker CD206^+^ positive cells was decreased through RBM15 deficiency, which was abolished by the ectopic expression of ITGBL1. In parallel, the lack of RBM15 could markedly restrain M2 markers (CD163, CD206, and IL-10), which was partly overturned after ITGBL1 co-transfection (Figure 7B and 7C). After co-culturing CD8^+^T cells and treated Lovo and SW620 cells, CFSE staining presented that RBM15 silencing-induced CD8^+^T cell proliferation rate promotion was significantly abrogated by ITGBL1 upregulation (Figure 7D). Synchronously, flow cytometry analysis displayed that RBM15 downregulation dampened the proportion of CD8^+^ T cell apoptosis, and these influences were partly ameliorated through ITGBL1 overexpression (Figure 7E). Overall, these data suggested that RBM15 knockdown could inhibit COAD immune response by regulating ITGBL1.

## Discussion

Although encouraging progression has been made in the efficiency of current immunotherapies, there is still an enormous group of patients who do not have a durable response owing to acquired resistance.^22^ Of note, these mechanisms might be associated with dysfunction of effector cells, which could be surmounted by immune checkpoint inhibitors (ICIs) and the production of immunosuppressive TMCs.^23^ As a member of the EGF-like protein family, ITGBL1 expression was upregulated in anti-PD-1 resistant patients.^8^ Further study suggested that ITGBL1 could help melanoma cells escape immune surveillance by hindering NK cell cytotoxicity and counteracting the beneficial effects of anti-PD-1 treatment.^9^ Furthermore, ITGBL1 is a secreted protein that regulates integrin activity during chondrogenesis.^24^ It has been reported that integrins adjust cell morphology, polarization, motility, adhesion, and signal transduction.^25^ Therefore, ITGBL1 was selected for further research.

In fact, several investigations have shown that ITGBL1 induces cell migration, invasion, and adhesion in different cancers,^26^ containing CRC.^12^ Herein, public databases analyzed the expression changes of ITGBL1 at different stages of COAD and determined that ITGBL1 content was related to the metastasis of COAD. Functional experiments displayed that the downregulation of ITGBL1 repressed COAD cell proliferation and metastasis. Previous studies have suggested that lymphocytes infiltrating into the tumor, such as tumor-associated macrophages and regulatory T cells, can mediate the immunosuppressive TME and help the tumor cells to achieve immune escape, thereby promoting the malignant progression of the tumor.^27,28^ Considering that ITGBL1 was a new immunomodulator that expedited melanoma progression by suppressing the cytotoxicity of NK cells, we further analyzed the relationship between ITGBL1 and immune infiltration in COAD cells. Based on TIMER and GEPIA database analysis, we found that ITGBL1 has a close correlation with immune-infiltrating cells and M2-type macrophage markers. Furthermore, there was a positive correlation between ITGBL1 and PD-L1 expression in COAD patients. Hence, it is hypothesized that ITGBL1 may modulate COAD progression by affecting the interaction between immune cells and malignant tumor cells. Herein, our data exhibited that CM from COAD cells could activate and drive the differentiation of in vitro THP-1 monocytes toward M2 phenotype macrophages, which was partly alleviated by ITGBL1 knockdown. Meanwhile, the lack of ITGBL1 could improve T-cell proliferation and reduce T-cell apoptosis in COAD cells. These findings indicate that ITGBL1 could regulate immune function, and its deficiency could restrict COAD cells from escaping immune surveillance by inhibiting M2 macrophage polarization and increasing T-cell populations. In other words, inhibition of ITGBL1 could improve COAD response to immunotherapies.

Regarding the molecular mechanism, emerging evidence has suggested that m6A modification plays an essential role in mRNA stability, pre-mRNA splicing, and translation.^29^ As a common m6A methyltransferase, RBM15 is primarily localized in the nucleus and can bind to RNA by interacting with spliceosome components.^30^ Beyond that, RBM15 was highly expressed in CRC patients and cells, and could bind the m6A-methylation complex to regulate KLF1 mRNA formation.^19^ Another recent study exhibited that RBM15 could boost CRC cell proliferation and metastasis by m6A-mediated modification of MyD88 mRNA.^18^ Herein, our data validated that ITGBL1 was a possible downstream target of RBM15-mediated m6A modification in COAD cells. Moreover, RBM15 could enhance ITGBL1 expression in COAD cells. Furthermore, RBM15 silencing-mediated COAD cell growth inhibition and immunosuppression were partly abolished by ITGBL1 upregulation, validating the RBM15/ITGBL1 regulatory mechanism. However, our research has some shortcomings. First, a patient-derived xenograft (PDX) COAD model should be established in vivoto further validate these novel regulatory mechanisms. Second, ITGBL1 expression was determined in a limited number of tissue samples. More respondents should be recruited in future studies.

**Figure 8.**
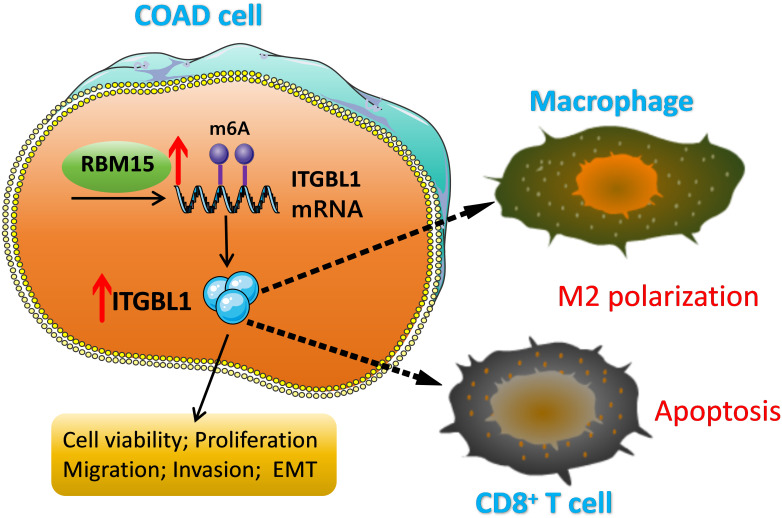
m6A methylase RBM15-mediated upregulation of ITGBL1 mRNA stability could boost COAD cell proliferation, migration, invasion, EMT, and immune escape.

In summary, these current works elucidated that RBM15 could facilitate COAD progression and immune escape by improving ITGBL1 mRNA stability (Figure 8), providing a new avenue of therapy for COAD.

## Figures and Tables

**Table 1.** Correlations Between ITGBL1 Expression and Various Clinicopathological Characteristics of Colon Adenocarcinoma

Clinical Feature	n	ITGBL1		*P*
		High	Low	
Age (years)		23	22	.4578
≥60	23	13	10	
<60	22	10	12	
Gender				.6661
Male	24	13	11	
Female	21	10	11	
Lymph node metastasis				.2917
N0	15	6	9	
N1-N2	30	17	13	
Distant metastasis				.0173*
Without	29	11	18	
With	16	12	4	
Tumor size (cm)				.2989
>5	28	16	12	
≤5	17	7	10	
Clinical stage				.0421*
I-II	14	4	10	
III-IV	31	19	12	

ITGBL1, integrin β-like 1 protein; N0, there is no cancer in nearby lymph nodes; N1-N2, the number and location of lymph nodes that contain cancer.

**Table 2.** Primers Sequences Used for PCR

Name		Primers for PCR (5’-3’)
RBM15	Forward	CGAGATAGGAAGCACCGGAC
	Reverse	CCCCATCCTGTTTCTGGGAC
ITGBL1	Forward	GTGGCAGGTGTAAGTGTGATA
	Reverse	CAGCCTTGCAGTAGCAGTTTC
PD-L1	Forward	ATTTGCTGAACGCCCCATAC
	Reverse	TCCAGATGACTTCGGCCTTG
CD163	Forward	AGTCTGCTCAAGATACACAGAAA
	Reverse	TGCTCCTCCTGGGGTAGAAA
CD206	Forward	CCAAACGCCTTCATTTGCCA
	Reverse	ACCTTCCTTGCACCCTGATG
IL-10	Forward	TCTCCGAGATGCCTTCAGCA
	Reverse	TCACATGCGCCTTGATGTCT
β-actin	Forward	CCGCGAGAAGATGACCCAG
	Reverse	GATAGCACAGCCTGGATAGCA

IL-10, interleukin-10; ITGBL1, integrin β-like 1 protein; PD-L1, programmed death ligand 1; RBM15, RNA-binding motif protein-15.

## Data Availability

The analyzed data sets generated during the present study are available from the corresponding author upon reasonable request.
